# When things go wrong with you, it hurts me too: The effects of partner’s employment status on health in comparative perspective

**DOI:** 10.1177/0958928720963330

**Published:** 2021-01-18

**Authors:** Anna Baranowska-Rataj, Mattias Strandh

**Affiliations:** Umeå University, Sweden

**Keywords:** couples, family, job separations, social policies, spillover effects, unemployment

## Abstract

The effects of changes in employment status on health within couples have attracted increasing attention. This paper contributes to this emerging research by investigating whether the impact of a partner’s employment status on individual self-rated health varies systematically across countries with varying decommodification levels. We use longitudinal data from the European Union Statistics on Income and Living Conditions (EU-SILC) and hybrid models. We find that a change in an individual’s employment status may affect the health not just of the person who experiences this transition, but that of his or her partner. The likelihood that such a spillover will occur varies across countries with different decommodification levels. The negative effects of a partner’s employment status on self-rated health are observed when the generosity of welfare state support is limited. The moderating effects of financial support from the state are not very strong, though. They are not robust across all our models and do not extend to all the dimensions of the generosity of welfare state support.

## Background

The growing volatility of labour markets in Europe and United States has raised concerns about the consequences of job losses for population health. Employment is not only the main source of income for the majority of people of working age, it has also been shown to be vital for developing social contacts, sharing goals and purposes with others, defining important aspects of personal status and identity (Jahoda, 1981). Individuals who are not involved in paid work are deprived of these benefits. The lack of them may lower their self-esteem and trigger stress and anxiety. A lack of a job may also leave traces on physical health, due to accumulation of mental health problems over a long period of time and as a result of health-related behaviours adopted after stopping paid work (Pampel et al., 2010). Thus, a lack of gainful employment can lead to a deterioration in an individual’s mental and physical health (Jahoda, 1981; Pearlin et al., 1981). While a number of studies have examined the associations between employment status and health (McKee-Ryan et al., 2005; Paul and Moser, 2009; Wanberg, 2012), these associations have so far been investigated mainly from an individual perspective. However, a change in employment status may affect not only the individual who experiences the transition, but also the people in his or her immediate social environment (Brand, 2015; Ström, 2003), and especially his or her partner.

Partnership plays a crucial role in psychological and health-related functioning (Carr and Springer, 2010; Rook et al., 1991), and thus constitutes a relevant context for investigating the extent to which important life course transitions such as changes in employment status are related to health. On the one hand, being in a partnership can buffer the effects of changes in employment status, because the partner can provide financial and emotional support (Tattarini et al., 2018). On the other hand, being in a partnership means combining two otherwise separate life plans, responsibilities and experiences. This thus increases an individual’s exposure to risk factors for ill health through emotional closeness to and economic interdependence with another person. A growing number of studies have therefore turned their attention to the consequences of changes in employment status from the perspective of both men and women in heterosexual relationships.

The aim of this paper is to examine the impact of changes in one partner’s employment status on the self-rated health of the other partner.^1^ This topic has recently attracted a growing attention (Inanc, 2018; Kim and Do, 2013; Marcus, 2013; Mendolia, 2014; Nikolova and Ayhan, 2019). We contribute to this strand of research by looking at how the health effects of changes in a partner’s employment status differ across European countries. Previous research has shown that social policies moderate the health effects of unemployment (Bambra and Eikemo, 2008; O’Campo et al., 2015). We build on this research and examine whether financial support from the state protects a partner’s health as well.

The contribution of this study to the existing research is as follows. We add to the still limited, but growing empirical evidence regarding the effects on health of changes in the partner’s employment status. In our analyses, we use longitudinal data from the European Union Statistics on Income and Living Conditions (EU-SILC). We employ panel data methods that reduce the bias that may result from the unobserved heterogeneity among couples in which one of the partners experiences a change in employment status. Whereas many surveys interview only selected representatives of households, the EU-SILC takes a multi-actor perspective in which information on all adult household members is collected. Hence, this dataset provides us with information about both co-resident partners (married or in informal cohabitation), which can be used to make inferences regarding the health effects of a partner’s employment status. Finally, as the EU-SILC includes data from a large number of countries, we take advantage of the variation in policy settings to extend the knowledge on how policies may shelter families from the consequences of changes in employment status. Hence, we apply an international comparative perspective to examine how changes in individuals’ employment status affect the health of the partner and how such effects are intensified or buffered under different social policy contexts.

### An individual versus a couple-centred perspective

The literature on the effects of employment on health has indicated that having a job can serve several important functions (Jahoda, 1981; Nordenmark and Strandh, 1999; Strandh, 2000). Paid work provides individuals with income, which is essential to both mental and physical health. In addition to enabling them to satisfy their physical needs, having an income can cause people to feel that they have control over their own life and the ability to plan ahead. In addition, employment satisfies a number of psychological needs, because working helps people structure their time, enhances their social status and gives them a sense of identity. Moreover, employment provides people with access to social contacts, externally generated goals and collective purpose, and opportunities to use skills and develop competences. Deprivation of most important human needs after a job loss not only impairs mental health but also leaves traces on physical health (Brand, 2015; McKee-Ryan et al., 2005). However, previous research points to differences in how a lack of gainful employment affects the health of men and women. Men and women are socialized towards different roles, with women being held responsible for household duties and men for achieving high economic status (Eagly and Wood, 2016). These socialization processes have implications for how changes in employment status are experienced across gender. Given that men’s perception of masculinity and self-esteem often are determined by the capacity to support one’s family financially (Donaldson, 1993), it is perhaps not surprising that ‘masculine identity is intricately linked to having a job’ (Paul and Moser, 2009), although there is no consensus that these differences are universal across all societal contexts (McKee-Ryan et al., 2005).

Previous research on changes in employment status and health examined this relationship from an individual perspective. However, most recent contributions to this literature have stressed that human health trajectories do not develop in isolation from one another, and that a combination of information on the multiple actors within each individual’s social environment is needed to get a full picture of the effects of employment status on health (Brand, 2015). In particular, it has been shown that partnerships constitute micro-ecosystems in which both mutual support and tensions related to financial and psychological resources are of paramount importance (Carr and Springer, 2010). As resources tend to be shared within each household, the financial consequences of a change in employment status may be harmful not just for the individual who experiences this transition, but for his or her partner. Indeed, previous research has shown that economic deprivation and strain are associated with a deterioration in personal relationships (Conger et al., 1990; Voydanoff, 1990). There is also a large body of evidence on so-called ‘spillover effects’; that is, the within-person transmission of feelings and emotions across different life domains, which can occur despite the physical and the temporal boundaries between a person’s professional and family lives (Staines, 1980). Distress can spill over from the work-related domain to the home-related domain, and then cross over to the people who are most closely related to the individual experiencing the distress; and especially to the individual’s partner (Bakker et al., 2009; Howe et al., 2004). These crossover effects may operate through empathy; that is, the sharing of the partner’s emotional state. The transmission of distress between the partners may also operate in a different way: the changes in the employment status of one of the partners may lead to emotional reactions and behaviours that place a burden on the other family members, which can in turn cause their health outcomes to deteriorate (Marcus, 2013; Rook et al., 1991).

Previous research suggested that the health responses to changes in employment status may depend on which of the partners makes the transition out of employment. Given differences in wages between men and women, the financial consequences of job losses in heterosexual couples are larger when male partners lose the source of earnings as compared to when it happens to women. The diffusion of the health effects of transition out of employment may be also stronger among women because women are more vulnerable to negative events that occur to others in their social network. In contrast, previous research has seen men as more vulnerable to negative events that occur to themselves (Simon, 2014).

### The role of the welfare state

Social policies represent powerful forces that shape both exposures and responses to major life course risks, such as losing a job. Some of the welfare state’s key functions are: regulating the relationship between the market and the individual, satisfying needs of individuals, offering them greater autonomy as well as shaping public opinions and conveying normative messages (Grönlund and Öun, 2010). One of the key policy dimensions is de-commodification, or the degree to which individuals have to rely on income from paid work (Esping-Andersen, 1999). By providing income necessary to cover the basic expenses, and making individuals less dependent on market forces, welfare state support decreases distress related to experiences of economic hardship, or anticipation thereof, and hence reduces the risk of health problems (Muntaner et al., 2011). The support from the welfare state also decreases economic insecurity and provides better opportunities to decide freely how to organize professional and private life. This in turn contributes to the feeling of agency and control over one’s own situation, which in turn positively affects health and wellbeing (Fryer, 1986). Finally, social policies convey normative messages about what is considered as socially acceptable for an individual and for a couple when it comes to labour market participation. Specifically, the eligibility rules and generosity of social policies may reduce or strengthen stigma related to lack of paid work (Voßemer et al., 2017; Wulfgramm, 2014). Hence, the link between employment status and health may depend on institutional settings.

A large body of evidence confirms the protective role of welfare state support for health and wellbeing of the unemployed (O’Campo et al., 2015). Comparative welfare state research indicates that welfare state support plays a shielding role not only for the contemporaneous beneficiaries but also for the whole working population (Ferrarini et al., 2014). It has been argued that income schemes supporting the unemployed may be seen as a ‘collective resource’ which reduces the negative consequences of employment insecurity and economic uncertainty among those who still have their jobs (Sjöberg, 2010). In this paper, we argue that the availability of financial support provides financial relief not only for the individuals who experience changes in employment status but also for their partners. In addition, in societal contexts where individuals without jobs may maintain a decent life standard, and hence enjoy greater agency and do not feel stigmatized, the crossover effects described in the previous section of this paper may be substantially reduced.

### Review of empirical studies

The effects of changes in employment status on health within couples have received increasing attention. In a study focused on heterosexual couples aged 50–60 in the US, Siegel et al. (2003) found no evidence that the husband’s job loss had a statistically significant effect on the wife’s mental health. In studies conducted using data on heterosexual couples in Germany, Marcus (2013) observed that the job loss of a partner had greater negative effects on mental health if the partner was male than if the partner was female and Nikolova and Ayhan (2019) have shown that the effects on subjective wellbeing were more persistent among women. A similar result was obtained by Kim and Do (2013) in a study focused on subjective wellbeing among Korean heterosexual couples. In studies using data for the UK, Mendolia (2014) and Inanc (2018) found that heterosexual couples in which the husband had experienced a job loss were more likely to experience poor mental health and reduced psychological wellbeing. A similar conclusion has been reached by Bubonya et al. (2017) for Australia.

In sum, the few existing studies on how a change in an individual’s employment status affects the partner have been focused on specific country cases and hence provide results that cannot be generalized to all kinds of societal contexts. In this paper, we add to this research by providing systematic evidence on how social policies may shield heterosexual couples from the potentially negative consequences of changes in employment status experienced by partners.

## Research design

In this study, we use longitudinal data and methods. We employ panel data from the harmonized EU-SILC database, which covers 28 European countries over the 2004–2015 period.^2^ As the EU-SILC is a household survey, it provides information on the employment status of each partner in a couple, which is crucial for answering our research questions.^3^ The overall response rates vary from 95% in Romania to 60% in Denmark (Wolff et al., 2010). Because the EU-SILC has a panel design, our analyses can draw upon repeated observations. In most of the countries, the EU-SILC has a rotational panel component in which each individual is observed for 4 years. The specific panel design is somewhat different in certain countries, including in France (nine rotational groups), Norway (eight rotational groups), and Luxembourg (a traditional panel). Across the participating countries the share of re-interviewed persons is around 83% (Iacovou et al., 2012). Thus, the EU-SILC is the largest European survey providing harmonized panel data that can be used to follow over time individuals embedded in different institutional contexts (Arora et al., 2015).

While the EU-SILC includes identification numbers that track individuals across subsequent survey waves, the personal identification numbers are reused when a rotational panel finishes and a new panel subsample is introduced. In order to avoid duplicates, we constructed our sample by choosing three non-overlapping sample components following individuals in the 2004–2007, 2008–2011 and 2012–2015 panels. We restricted the sample to individuals aged 18–50 and their partners,^4^ who did not participate in education.^5^ We compare how self-rated health of partners in these couples changes depending on whether or not one of the partners experiences a change in employment status. While our sample is restricted to people aged 18–50, we did not condition the sample on a partner’s age. Information on a partner’s employment status is included even if a partner was aged 16–17 or over age 50. Due to asymmetry in the ages of the partners (on average, the male partner is older than the female partner) and the way we imposed the age restriction, the sample sizes of men and women differ. In our analysis, we use data for 114,422 women and 99,722 men.

Our key dependent variable is constructed based on the respondents’ self-assessments of health. The respondents rated their health using a five-category scale, with values ranging from very good (1) to very bad (5). To make the interpretation of the results easier, in the analyses the scale was reversed so that higher ratings indicate better health. Our key explanatory variable captures the transitions out of employment among partners of individuals observed in our data. For each individual in our sample, a time-varying variable measuring the partner’s employment status distinguishes between employment, unemployment and inactivity. The category of employed includes individuals working full-time and part-time as well as self-employed. Unemployment is defined as not having a job and searching for one, and it is self-assessed. The group of the economically inactive comprises individuals who consider themselves unfit for work, who have given up business, or are fulfilling domestic tasks and care responsibilities. A vast number of studies on employment status and health have omitted this category and focused instead on comparing the employed with the unemployed. However, studies which do distinguish between unemployment and economic inactivity show that the association with poor health may actually be even stronger in the case of the latter labour market status (Popham and Bambra, 2010; Popham et al., 2012).

The control variables include individual age and educational attainment (with the following categories: elementary or lower education, lower secondary education, upper secondary education, postsecondary education and tertiary education). Additionally, we control for marital status and distinguish between individuals who were cohabiting and married. We also include a variable operationalising individual employment status, which has the same categories as the variable measuring partner’s employment status. Moreover, we control for the presence of children up to age 16. We also include fixed effects for survey years and for countries. The sample structure is presented in Table A1 in the Annex.

We link our micro-data from EU-SILC with macro-level measures of de-commodification. We use measures of OECD net replacement rates to capture generosity of welfare state support for people losing jobs. Net replacement rates show the proportion of net income that is maintained after job loss. The measures available in OECD Tax and Benefit Database take different values according to worker’s prior income and length of elapsed unemployment, eligibility for social assistance, the presence of a second earner and children in the household. We use net replacement rates that are relevant in the initial phase of unemployment for a worker with earnings equal to an average wage in his or her country. Since we do have individual-level information on household composition, we link specific indicators of net replacement rates conditional on employment status of a person whose partner became unemployed and conditional on whether or not a couple has children aged 16 or under.

We carried out a set of additional analyses. The OECD indicator of net replacement rates can include or exclude housing benefits and social assistance. We tested the robustness of our results by using an alternative version of this indicator. In addition, we used replacement rates derived from the SPIN database (Doctrinal et al., 2015), which consider unemployment benefits for workers earning above 33% of the average wage. Unfortunately, this database has not been updated during years 2012–2015, and hence when using this indicator we lose a substantial portion of our data. While net income replacement rates are seen as the critical feature of the decommodification index (Scruggs and Allan, 2006), other dimensions of decommodification also deserve attention. Studies comparing different aspects of welfare state generosity may show different results (McKee-Ryan et al., 2005; O’Campo et al., 2015). In order to test whether the social protection coverage of the unemployed has similar effects as replacement rates, we estimated models with interactions between employment status and the proportion of beneficiaries of out-of-work income support among the unemployed in years 2004–2015 from the Eurostat’s Labour Market Policy Indicators Database. Finally, social policies not only determine the importance of gainful employment for individual wellbeing, but also structure the gender relations. Countries with more generous financial support from the state may create conditions that ensure more equality between men and women. To test whether our results are robust after accounting for macro-level factors related to gender relations, we carried out additional analyses using the Global Gender Gap Index (Lopez-Claros and Zahidi, 2005), compiled by the United Nations Development Program and the World Bank as well as executive opinion survey data collected by the World Economic Forum. The index captures women’s economic participation, economic opportunity (including the impact of laws on hiring practices), political participation, educational attainment and health and wellbeing. Table A2 in the Annex presents the distribution of all the macro-level variables used in our analysis.

Based on the EU-SILC data, we estimated hybrid models with an ordered logit link (Bell and Jones, 2015). Hybrid models use a flexible modelling approach that separates within- and between-person effects and allows for the consistent estimation of the effects of time-varying characteristics in a manner similar to that of fixed-effects models.^6^ This is accomplished by including both the deviations from the person-specific means of time-varying characteristics and the person-specific means of these characteristics in the set of the model covariates. These transformations are applied to both individual-level variables (specifically, marital status, parenthood status and individual employment status are both time-varying and potentially endogenous and hence were demeaned) and to the indicators of policies. The inclusion of the person-specific means of time-varying characteristics picks up any correlation between these variables and the unobserved random effects. This modelling approach reduces the possible bias resulting from unobserved heterogeneity among the individuals in our sample, which is important because previous research has indicated that the risk of exit from employment is not distributed randomly in the working-age population, and varies depending on health status (Brand, 2015). The use of hybrid models has also been shown to be more appropriate than commonly used fixed effects models in the context of panel data with short time dimension and unbalanced structure (Bell and Jones, 2015; Wooldridge, 2009). This is the case in our study, where most couples are observed for less than four survey waves and the number of observation points varies across individuals. While our models control for unobserved heterogeneity in a manner similar to that of fixed-effects models (Bell and Jones, 2015; Schunck and Perales, 2017), one should still keep in mind that there may be time-varying factors that bias our results. We estimated separate models for men and women.

## Empirical results

In the first step, we present how individual and partner’s employment status affects self-rated health among women and men across European countries. The results in Table 1 present the log odds ratio from hybrid models of self-rated health for pooled data. According to results from Model 1, among women, we do not observe any statistically significant effects of transition into unemployment or inactivity on self-rated health. The same holds for the effects of a partner’s unemployment and inactivity on a woman’s self-rated health. According to the results from Model 2, changes in a man’s own employment status had a strong effect on self-rated health, but the employment status of his partner had no negative impact on this outcome. Actually, the transition of a female partner into inactivity is positively related to self-rated health.

**Table 1.** Self-rated health according to the partner’s employment status: results from hybrid effects ordered logistic models.

	Model 1 women	Model 2 men	Model 3 women	Model 4 men	Model 5 women	Model 6 men	Model 7 women	Model 8 men	Model 9 women	Model 10 men
Effects of control variables										
Age	−0.089***	−0.095***	−0.088***	−0.093***	−0.088***	−0.093***	−0.086***	−0.094***	−0.092***	−0.097***
	(0.001)	(0.001)	(0.001)	(0.001)	(0.001)	(0.001)	(0.001)	(0.002)	(0.001)	(0.001)
Education: (ref. lower secondary)									−0.411***	−0.265***
Elementary or lower	−0.227***	−0.092**	−0.208***	−0.059	−0.194***	−0.056	−0.119***	0.028	(0.036)	(0.037)
	(0.035)	(0.036)	(0.035)	(0.036)	(0.035)	(0.036)	(0.041)	(0.042)	0.327***	0.200***
Upper secondary	0.285***	0.159***	0.247***	0.139***	0.257***	0.134***	0.217***	0.108***	(0.023)	(0.023)
	(0.023)	(0.023)	(0.023)	(0.023)	(0.023)	(0.023)	(0.028)	(0.028)	0.432***	0.272***
Post secondary	0.338***	0.237***	0.281***	0.216***	0.287***	0.211***	0.220***	0.204***	(0.042)	(0.047)
	(0.042)	(0.046)	(0.042)	(0.046)	(0.042)	(0.046)	(0.049)	(0.054)	0.994***	0.930***
Tertiary	1.063***	1.036***	1.022***	1.008***	1.030***	1.000***	1.026***	1.034***	(0.026)	(0.027)
	(0.026)	(0.026)	(0.026)	(0.026)	(0.026)	(0.026)	(0.031)	(0.032)	−0.092***	−0.097***
Effects of within-differences										
Married	0.093*	−0.085*	0.085*	−0.096*	0.085*	−0.097*	0.064	−0.099	0.068	−0.133***
	(0.048)	(0.050)	(0.048)	(0.050)	(0.048)	(0.050)	(0.059)	(0.061)	(0.050)	(0.051)
The presence of children	0.013	−0.075**	0.069**	0.014	0.079***	0.027	−0.002	−0.064*	0.024	−0.077**
	(0.029)	(0.030)	(0.029)	(0.031)	(0.029)	(0.031)	(0.035)	(0.037)	(0.029)	(0.031)
Unemployed	−0.041	−0.197***	−1.226***	−1.713***	−1.405***	−1.759***	−0.402***	−0.381**	−0.121***	−0.258***
	(0.026)	(0.034)	(0.143)	(0.183)	(0.167)	(0.225)	(0.117)	(0.162)	(0.045)	(0.059)
Inactive	−0.042	−0.493***	−1.173***	−1.936***	−1.138***	−1.600***	−0.218	−0.138	0.049	−0.413***
	(0.029)	(0.057)	(0.160)	(0.281)	(0.192)	(0.357)	(0.146)	(0.283)	(0.052)	(0.100)
Partner unemployed	−0.020	−0.005	−0.371**	−0.667***	−0.344*	−0.731***	−0.119	−0.292**	−0.039	−0.060
	(0.032)	(0.027)	(0.164)	(0.173)	(0.198)	(0.196)	(0.152)	(0.121)	(0.054)	(0.048)
Partner inactive	−0.049	0.076**	−0.615**	−0.406**	−0.398	−0.520**	−0.229	−0.218	−0.234***	0.141**
	(0.050)	(0.031)	(0.253)	(0.194)	(0.303)	(0.218)	(0.250)	(0.151)	(0.088)	(0.056)
Net replacement rates (NRR)			−0.022***	−0.035***	−0.024***	−0.035***	−0.014***	−0.012***		
			(0.002)	(0.002)	(0.002)	(0.002)	(0.002)	(0.002)		
Unemployed × NRR			0.014***	0.017***	0.017***	0.018***	0.006***	0.002		
			(0.002)	(0.003)	(0.002)	(0.003)	(0.002)	(0.003)		
Inactive × NRR			0.013***	0.016***	0.013***	0.012**	0.003	−0.006		
			(0.002)	(0.004)	(0.003)	(0.005)	(0.002)	(0.005)		
Partner unemployed × NRR			0.005**	0.009***	0.004*	0.010***	0.002	0.005**		
			(0.002)	(0.002)	(0.003)	(0.003)	(0.003)	(0.002)		
Partner inactive × NRR			0.008**	0.006**	0.005	0.008***	0.003	0.004*		
			(0.003)	(0.003)	(0.004)	(0.003)	(0.004)	(0.003)		
Unemployment benefit coverage (COV)									0.003*	0.005***
									(0.001)	(0.001)
Unemployed × COV									0.002**	0.002
									(0.001)	(0.001)
Inactive × COV									−0.002*	−0.002
									(0.001)	(0.002)
Partner unemployed × COV									0.001	0.002*
									(0.001)	(0.001)
Partner inactive × COV									0.005**	-0.002
									(0.002)	(0.001)
Effects of between-differences										
Married	0.004	−0.048**	−0.054**	−0.089***	−0.052**	−0.088***	−0.123***	−0.156***	0.151***	0.108***
	(0.024)	(0.024)	(0.024)	(0.024)	(0.024)	(0.024)	(0.031)	(0.031)	(0.025)	(0.025)
The presence of children	0.444***	0.181***	0.500***	0.280***	0.513***	0.296***	0.496***	0.221***	0.383***	0.123***
	(0.019)	(0.019)	(0.020)	(0.020)	(0.020)	(0.020)	(0.023)	(0.023)	(0.019)	(0.019)
Unemployed	−0.789***	−1.918***	−1.632***	−4.934***	−1.665***	−5.251***	−0.348***	−3.093***	−0.763***	−1.947***
	(0.027)	(0.039)	(0.134)	(0.194)	(0.160)	(0.249)	(0.107)	(0.169)	(0.043)	(0.063)
Inactive	−0.668***	−3.627***	−2.881***	−7.023***	−3.094***	−6.708***	−1.009***	−4.023***	−0.015	−2.977***
	(0.029)	(0.063)	(0.144)	(0.274)	(0.172)	(0.362)	(0.148)	(0.309)	(0.048)	(0.105)
Partner unemployed	−1.059***	−0.362***	−1.689***	0.353**	−1.555***	0.732***	−1.311***	0.156	−1.079***	−0.389***
	(0.036)	(0.027)	(0.180)	(0.172)	(0.226)	(0.196)	(0.156)	(0.109)	(0.058)	(0.043)
Partner inactive	−0.948***	−0.001	−3.340***	0.188	−3.374***	0.134	−1.978***	−0.243	−0.538***	0.371***
	(0.053)	(0.029)	(0.253)	(0.178)	(0.322)	(0.205)	(0.275)	(0.149)	(0.089)	(0.050)
Net replacement rates (NRR)			−0.034***	−0.031***	−0.040***	−0.032***	−0.020***	−0.018***		
			(0.001)	(0.001)	(0.001)	(0.001)	(0.001)	(0.001)		
Unemployed × NRR			0.009***	0.040***	0.010***	0.045***	−0.008***	0.014***		
			(0.002)	(0.003)	(0.002)	(0.004)	(0.002)	(0.003)		
Inactive × NRR			0.031***	0.047***	0.033***	0.041***	0.007***	0.008		
			(0.002)	(0.004)	(0.003)	(0.005)	(0.003)	(0.005)		
Partner unemployed × NRR			0.009***	−0.009***	0.007**	−0.014***	0.002	−0.009***		
			(0.003)	(0.002)	(0.003)	(0.003)	(0.003)	(0.002)		
Partner inactive × NRR			0.035***	−0.003	0.034***	−0.002	0.019***	0.005**		
			(0.004)	(0.002)	(0.004)	(0.003)	(0.005)	(0.003)		
Unemployment benefit coverage (COV)									0.016***	0.015***
									(0.000)	(0.000)
Unemployed × COV									−0.000	0.004***
									(0.001)	(0.001)
Inactive × COV									−0.015***	−0.016***
									(0.001)	(0.002)
Partner unemployed × COV									0.003**	0.001
									(0.001)	(0.001)
Partner inactive × COV									−0.009***	−0.008***
									(0.002)	(0.001)
Residual variance	6.091***	5.394***	6.000***	5.292***	5.993***	5.283***	6.028***	5.329***	5.724***	5.060***
	(0.055)	(0.054)	(0.055)	(0.053)	(0.055)	(0.053)	(0.067)	(0.065)	(0.054)	(0.053)
Person-observations	301744	260862	301744	260862	301744	260862	201620	175377	285328	247012

EU-SILC. Notes: *p < 0.05, **p < 0.01, ***p < 0.001. Self-rated health ratings: 1 = very bad, 5 = very good. Effects of ancillary parameters omitted.

In the next step, we explore heterogeneity of these effects and we investigate whether their magnitude is related to de-commodification. In Models 3 and 4 we include interactions between individual and partner’s employment status with indicators measuring net replacement rates. These results reveal that among women, the effects of changes in both own and partner’s employment status are related to poorer self-rated health when welfare state support is non-existent, that is, at net replacement rates equal to zero. The effects of partner’s employment status are weaker than the effects of own employment status and they are statistically significant only at the 5% level. The increase in net replacement rates reduces the magnitude of the effects of both own and partner’s employment status on women’s self-rated health. Again, one should note that the interaction effects between partner’s employment status and net replacement rates are not strong and they are statistically significant at the 5% level. Among men, we observe a similar pattern. If net replacement rates are equal to zero, transitions into unemployment or inactivity experienced by them or by their partners are related to poorer self-rated health. An increase in net replacement rates reduces the negative effects of own or partner’s transitions into unemployment. However, again, these moderating effects are much weaker when it comes to partner’s employment status and they are statistically significant at 5% in the case of transitions into inactivity.

Regarding our robustness checks, Models 5 and 6 replicate Models 3 and 4 with indicators of net replacement rates that take housing benefits into account. In these analyses, the effects that we previously observed among women whose partners become inactive lose statistical significance. For men, all the results remain quite similar. Analyses with indicators of net replacement rates derived from SPIN database (Model 7 and 8) cover a shorter period, which may be one reason why some of the results were no longer statistically significant. Analyses with indicators of coverage rates (Model 9 and 10) suggest that the effects of partner’s unemployment on women’s health tend to be less negative in countries where the coverage is higher, however the coefficients are really small and hence the positive effects of this dimension of social policy are negligible. Moreover, we do not observe any moderating effects of coverage among men.

The effects of the control variables are rather similar across model specifications. Age is shown to be associated with worse self-rated health, whereas educational attainment is found to be associated with better self-rated health. Compared to men in cohabiting partnerships, married men tend to report poorer health, while for women marriage is positively related to self-rated health in most specifications. The presence of children in a household is related to poorer self-rated health among men and has no statistically significant effect on women’s self-rated health (in most specifications).

The effects of between-person differences presented in Table 1 also merit reflection as they shed some light on the possible selection processes related to self-rated health of both individuals and their partners. Our results indicate selectivity of individuals who were more likely to make transitions out of employment. Moreover, individuals with poor health are overrepresented in the group with partners who have a higher propensity to make transitions out of employment. These selection effects are stronger among women than among men, suggesting that women with better self-rated health may be more likely to form partnerships with men who tend to have better labour market outcomes. Not accounting for these sources of bias would lead us to overestimate the impact of the partners’ employment transitions on individual self-rated health. We also observe that among women, the selection effects are relatively weaker in countries with higher net replacement rates. The opposite is true when it comes to self-rated health among men, whose partners become unemployed.

The effects of hybrid models presented in Table 1 may be difficult to interpret as they are measured on the log odds ratio scale. We therefore calculated marginal effects of the key explanatory variables, that is, partner’s unemployment and inactivity, based on Models 3 and 4. We focused on the probability of indicating very good health at two extreme values of net replacement rates: 30% and 80% of income.^7^ According to these results, if financial support from the state allows maintaining only 30% of net income after a job loss, the probability of reporting very good health decreases by 4 percentage points among women whose partners experienced a transition into unemployment. This marginal effect is rather small and statistically significant at 1% level. When the net replacement rates are at the level 80%, the marginal effect of a spouse’s unemployment reduces to almost zero percentage points and is not statistically significant. Similarly, the marginal effects of a partner’s transition into inactivity on woman’s chances to report very good health amounts to 6 percentage points and it is statistically significant at 1% level when the net replacement rates amount to 30%. However, this effect decreases to almost zero and becomes statistically not significant when the net replacement rates are at the 80% level. Regarding the marginal effects for men, we observe very similar patterns. If net replacement rates amount to 30%, the probability of reporting very good health decreases by 7 percentage points when a female partner becomes unemployed and 4 percentage points after a partner’s transition into inactivity. When net replacement rates amount to 80%, the probability of reporting very good health does not change due to a partner’s transition out of employment. Overall, this analysis confirms that the negative effects of changes in a spouse’s employment status on women’s self-rated health can be observed only in countries with very low levels of financial support from the welfare state.

Since previous research suggests that the effects of partner’s employment status may differ across gender, and yet our analyses do not reveal clear differences, we have carried out additional, more detailed analyses to get a better understanding why this might be the case. We have decomposed the variables capturing the effects of transitions into unemployment and inactivity according to the preceding labour market status. Our more detailed analyses presented in Table 2 distinguish between the effects of transition from employment to unemployment, remaining in unemployment in two consecutive years as well as making a transition from inactivity to unemployment. We also distinguish between the effects of a transition from employment to inactivity, remaining in inactivity in two consecutive years as well as making a transition from unemployment to inactivity. We make a similar distinction also for transitions into and out of employment. The reference category in this analysis is remaining in employment for two consecutive years. Similarly, as shown in previous research, male partners’ transitions from employment into unemployment as well as their repeated unemployment are related to poorer self-rated health among women. Some transitions out of unemployment experienced by a man are also related to poorer self-rated health by a woman. For instance, a transition from unemployment into inactivity has such an effect. Also, as compared to women whose partners are continuously employed, women with partners who work but experienced unemployment in the past report poorer self-rated health. In other words, partner’s re-employment, or more generally unstable career patterns, are related to poorer health as compared to continuous employment (which is the reference category in this analysis). Interestingly, among men, we do not observe any negative effects of past or contemporaneous unemployment experienced by their female partners. Overall, this analysis shows that the effects of partner’s labour market transitions are indeed gendered.^8^

**Table 2.** Self-rated health according to the differential changes in partner’s employment status – results from hybrid effects ordered logistic models.

	Model 11 Women	Model 12 Men
Effects of within-differences		
The effects of partner’s transition from (ref. repeated employment):		
Unemployment to employment	−0.058**	0.005
	(0.028)	(0.025)
Inactivity to employment	−0.110*	−0.059*
	(0.060)	(0.034)
Employment to unemployment	−0.085***	0.013
	(0.029)	(0.028)
Repeated unemployment	−0.066**	0.003
	(0.029)	(0.022)
Inactivity to unemployment	−0.092	−0.057
	(0.069)	(0.044)
Employment to inactivity	−0.048	0.036
	(0.053)	(0.032)
Unemployment to inactivity	−0.200***	0.033
	(0.053)	(0.031)
Repeated inactivity	−0.064	−0.050**
	(0.040)	(0.020)
Person-observations	301744	260862

EU-SILC. *Notes*. *p < 0.05, **p < 0.01, ***p < 0.001. Self-rated health ratings: 1 = very bad, 5 = very good. Effects of ancillary parameters omitted.

Gendered effects of employment status on health can be stronger in societies with a higher gender inequality (Strandh et al., 2013), which may coincide with more generous policies. In order to address this concern, we extended Models 3 and 4 presented in Table 1 by adding interactions between changes in employment status of an individual and his or her partner and the Gender Gap Index (see Table 3). We find that among women in countries with higher gender equality, the changes in employment status of male partners exert relatively weaker negative effects as compared to countries with less gender equality. At the same time, the impact of a female partner’s unemployment becomes negative if a country becomes more gender-equal. After adding interactions with GGI, the interactions between the employment status of a partner and indicators of decommodification are no longer statistically significant among women. One should note however that these models need to be interpreted with care as they include multiple cross-level interactions while also having a more limited time window than models presented in Table 1.

**Table 3.** Self-rated health according to the partner’s employment status: results from hybrid effects ordered logistic models including interactions with indicators of decommodification and gender equality.

	Model 13 women	Model 14 men
Effects of within-differences		
Partner unemployed	−1.011**	0.955**
	(0.474)	(0.390)
Partner inactive	−2.153***	−0.471
	(0.831)	(0.487)
Net replacement rates (NRR)	−0.013***	−0.027***
	(0.002)	(0.002)
Gender gap index (GGI)	−0.013***	−0.025***
	(0.004)	(0.005)
Partner unemployed × NRR	0.001	0.005*
	(0.002)	(0.002)
Partner inactive × NRR	0.006	0.005**
	(0.004)	(0.003)
Partner unemployed × GGI	0.012*	−0.018***
	(0.006)	(0.005)
Partner inactive × GGI	0.024**	0.002
	(0.011)	(0.006)
Person observations	275,231	237,586

EU-SILC. *Notes*. *p < 0.05, **p < 0.01, ***p < 0.001. Self-rated health ratings: 1 = very bad, 5 = very good. Effects of ancillary parameters omitted.

## Discussion and conclusion

Our paper contributes to the growing strand of research on the effects of changes in employment status within couples. Our study shows under what circumstances the adverse effects of transitions out of employment may affect the health not just of the person who experiences this transition, but also of his or her partner. We show that the degree to which the partner’s employment status matters for an individual’s self-rated health depends on the institutional context. We find that the effects of the partner’s employment status on self-rated health are stronger in contexts when the level of out-of-work benefits is low. The moderating effects of financial support from the state are not very strong, though. They are also not robust across all our models and do not extend to other dimensions of the generosity of welfare state support, such as the coverage of social protection.

Our additional analyses reveal interesting differences in the magnitude of the effects of partner’s employment status among men and women and also across countries with diverging policies supporting gender equality. We observe that as compared to men, women’s self-rated health is more strongly affected by a partner’s transition from employment into unemployment. This corroborates the results from previous studies (see, e.g. Inanc, 2018, Marcus, 2013; Mendolia, 2014; Nikolova and Ayhan, 2019). However, the negative health effects among women also emerge when their partners stop searching for jobs and become economically inactive.^9^ Overall, these findings are in line with arguments that women tend to be more affected than men by adverse events experienced by their family members (Simon, 2014). The asymmetry in how a partner’s unemployment affects the health of men and women may also be related to gender differences in the financial consequences of a job loss. As men continue to be more likely to be the main breadwinners within couples, the loss of their earnings may be more detrimental for households’ incomes than the loss of women’s earnings. This explanation is to some degree supported by our finding from additional analyses that in countries with greater gender equality, women are relatively less strongly affected by changes in their partner’s employment status. This suggests that conservative policies that impose higher gender wage gaps and barriers for occupational advancement of women make women more vulnerable to changes in the employment status of their partner.

The results from this study are relevant for public debates on social policies. The reforms in this area are usually based on a careful calculation of costs and benefits. However, much of the evaluation literature has focused on the re-employment effects of support directed at the unemployed, and has paid much less attention to the reduced wellbeing and potential public health costs (Rose, 2018). Moreover, policy evaluations are often based on a somewhat simplistic assumption that the individuals eligible to receive benefits under the policy are the only group who stand to gain from it (Smith and Sweetman, 2016). Recent studies have challenged this assumption by showing that the benefits from social policies may also be observed among employees, especially among those whose position on the labour market is particularly vulnerable (Ferrarini et al., 2014; Sjöberg, 2010). Our findings add to this research and indicate that an assessment of the benefits of programmes that target the unemployed should not be restricted to the participants of these programmes, but may also include the participants’ family members.

Our study has some limitations. The panel dimension of our data is short and we do not have the full information about the duration of the unemployment for both individuals and their partners. Previous research indicates that both financial consequences and the behavioural responses to the job loss may vary across the unemployment spell. Hence, the effects of a partner’s unemployment for individual health may also vary according to the time spent out of paid work. We nevertheless tried to capture this aspect in our additional analyses that look at the lagged employment status. Our microdata do not provide us with full information about working time, the type of employment contract and job tenure, and other factors that determine eligibility for cash benefits. Hence, some of the unemployed individuals in our data may not in fact be able to receive financial support from the state. Furthermore, while our data give us an opportunity for cross-country comparisons, we cannot explore all the possible moderating influences at the couple level. While this analysis focused on the role of financial support from the state, other policies supporting individuals who lose their jobs, may also be of importance. Active labour market policies are one example. Another limitation of our paper is that while our models control for fixed in time unobserved factors that may lead to a deterioration in health, they cannot handle endogeneity related to time-varying unobserved factors. Despite these limitations, we believe the insights from this study extend our knowledge on the consequences of adverse life course events. This paper adds empirical evidence showing that their influence may go beyond the individuals who experience them and reach their family members, especially in countries where public policies offer limited or no protection against societal risks and impose gender inequalities.

## Annex

**Table A1.** Sample structure: means and proportions.

	Women	Men
Self-rated health		
Very bad	0.5	0.4
Bad	3.1	2.6
Fair	15.6	14.5
Good	54.1	53.6
Very good	26.7	28.9
Age	38.8	39.8
Elementary or lower	6.0	6.2
Lower secondary	15.5	15.9
Upper secondary	43.1	47.7
Postsecondary	3.9	3.2
Tertiary	31.6	27.0
Married	83.6	82.3
Presence of children	64.4	68.1
Employed	70.8	91.2
Unemployed	15.8	6.5
Inactive	13.5	2.4
Partner employed	89.9	70.9
Partner unemployed	7.0	15.8
Partner inactive	3.1	13.2
Year	2010	2010
Observations	114,422	99,722
Person-observations	301,744	260,862

EU-SILC.

**Table A2.** Distribution of macro-level indicators across countries.

Country	Net replacement rates exc. housing benefits^a^			Net replacement rates inc. housing benefits^a^			Net replacement rates from SPIN database^b^			Out-of-work benefits coverage^c^			Gender Gap Index^d^		
	Mean	S.D.	Within-S.D.	Mean	S.D.	Within-S.D.	Mean	S.D.	Within-S.D.	Mean	S.D.	Within-S.D.	Mean	S.D.	Within-S.D.
AT	76	6.6	2.0	76	6.1	1.7	54	0.1	0.2	38	3.6	2	72	1.4	0.6
BE	70	5.8	1.2	70	5.8	1.2	55	2.1	0.7	102	11	7.7	74	2.5	1.3
BG	82	8.1	5.2	82	8.1	5.2	67	13.0	12.0	18	3.4	3	71	1.5	1.0
CY	78	3.6	0.8	78	3.6	0.8	68	0.2	0.3	33	1.3	1.8	65	0.5	0.7
CZ	75	8.0	2.0	76	6.8	1.8	52	3.4	1.1	25	4	2.6	68	0.5	0.3
DK	76	3.0	1.0	77	3.7	1.1	60	1.0	0.5	42	8.8	5.8	77	1.7	1.0
EE	71	5.6	2.2	71	5.6	2.2	55	0.3	0.3	16	4.8	3.5	71	1.4	1.1
ES	78	7.7	2.5	78	7.7	2.4	61	0.8	0.5	40	5.3	2.9	74	1.1	0.9
FI	76	6.9	3.4	77	4.5	2.1	54	0.8	0.8	60	5.1	3.3	83	1.9	0.6
FR	79	4.8	1.7	79	4.8	1.6	69	0.3	0.3	87	2.3	2.2	71	3.2	2.6
GR	51	12.0	3.0	53	11.0	3.4	36	5.6	2.4	n.a.	n.a.	n.a.	68	1.2	0.8
HR	76	7.7	2.1	76	7.7	2.1	n.a.	n.a.	n.a.	12	1.1	1.7	71	0.0	0.1
HU	70	8.7	4.0	71	7.9	3.8	51	5.1	4.3	31	8.6	2.7	67	0.7	0.5
IE	65	3.4	1.1	69	6.1	1.7	35	1.6	2.0	86	6.7	2.5	77	2.5	1.0
IS	75	5.2	3.3	76	3.9	2.4	52	6.2	3.5	n.a.	n.a.	n.a.	84	3.4	1.1
IT	75	5.2	2.1	75	5.2	2.0	52	1.4	0.6	17	3.7	1.3	68	2.3	0.8
LT	72	11.0	5.1	74	9.4	4.4	56	7.8	4.4	14	4.1	2.9	72	0.9	0.8
LU	90	2.5	0.9	90	2.5	0.8	79	0.4	0.4	48	16	12	70	2.6	1.6
LV	88	6.4	2.5	88	6.3	2.5	78	2.3	1.1	16	3.3	3.3	74	1.6	0.7
MT	55	5.4	1.3	58	4.4	1.8	39	1.4	1.6	32	9.1	7.4	67	0.4	0.3
NL	79	2.7	1.5	79	3.0	1.6	65	1.5	1.3	71	3.9	3.4	75	1.5	0.6
NO	80	3.2	1.1	80	2.8	0.9	56	0.3	0.3	25	4.8	5.3	83	1.6	0.8
PL	57	9.8	3.2	61	5.3	2.3	37	3.1	3.0	8.8	1.6	.79	70	1.2	0.4
PT	88	6.1	1.8	88	6.1	1.8	77	1.7	1.2	43	12	6	71	1.3	0.7
RO	59	10.0	2.5	59	10.0	2.5	44	1.8	4.7	12	1.7	1.6	69	0.6	0.2
SE	70	6.2	2.4	71	4.8	2.0	52	4.9	2.4	48	8.9	3.9	81	0.6	0.3
SI	81	4.8	2.1	82	3.7	1.9	58	3.6	2.7	21	9.7	6.5	71	3.0	1.3
SK	78	9.3	2.0	78	9.3	2.0	60	0.4	0.4	7.7	1.7	.88	68	0.3	0.3
UK	51	6.8	1.7	56	6.6	1.6	16	0.3	0.2	27	4	2.5	74	0.6	0.6

Within-S.D. are within-person standard deviations of macro-level variables, reflecting changes of values of these variables for individuals in the panel data.

a OECD Tax and Benefits Database.

b Out-of-Work Benefits dataset in SPIN database (Doctrinal et al., 2015).

c Eurostat.

d World Economic Forum.

## Notes

1. The partnership types considered in this paper include both married and consensual unions (that is, unmarried couples with or without a legal status).

2. The original EU-SILC panel dataset included data from 32 countries. The data were available under the contract with Eurostat no RPP 19/2019-EU-SILC.

3. The scope of information available for each partner within a couple depends on the country in EU-SILC data. In most countries in EU-SILC, all members aged 16 or over are asked to fill in a survey questionnaire. The exceptions to this rule are the following countries: Denmark, Finland, Iceland, the Netherlands, Norway, Sweden and Slovenia. In these countries, only one selected respondent per household receives a survey questionnaire, whereas socio-demographic information on the presence of other household members (most importantly, partners and their employment status) is obtained through administrative registers (Wolff et al., 2010).

4. From a technical point of view, the matching of partners in the analysed data is carried out using variables indicating an individual’s spouse/partner identification number available in EU-SILC data. Our analysis is restricted to partners of opposite sex. Observations when individuals are not partnered are excluded from the analysis.

5. We excluded person observations of pupils and students.

6. Fixed effects models show the within-person effect only. The advantage of hybrid models over fixed effects models is that they can present both time-invariant between-individual effects and time-varying within-individual effects (Schunck and Perales, 2017).

7. NRRs lower than 30% can be found for instance in Greece. NRRs equal or above 80% can be observed for instance in France.

8. This analysis also explains why the results presented in Table 1 do not show differences between men and women. Models that do not differentiate between the origins of labour market statuses, average the effects of entries and exits from unemployment. Because some transitions *out of* unemployment experienced by men have negative effects on self-rated health of their female partners, the effects of transitions *into* unemployment are cancelled out in our hybrid models presented in Table 1. Even though the results presented in Table 2 are more detailed, we cannot examine how they vary across countries because the number of origin-specific transitions per country becomes too restrictive and requires combining specific types of transitions into broader categories.

9. The transition from unemployment into inactivity could be interpreted as a ‘discouraged worker effect’. The concept of discouraged worker refers to withdrawing from job search because of losing hope to find a job. Previous research shows that for some unemployed looking for work has such a low expected pay-off, that they decide that spending time at home is more productive than spending time in job search (Blundell et al., 1998).
